# The Relationship Between Muscle Strength, Anaerobic Performance, Agility, Sprint Ability and Vertical Jump Performance in Professional Basketball Players

**DOI:** 10.2478/v10078-012-0016-6

**Published:** 2012-04-03

**Authors:** Utku Alemdaroğlu

**Affiliations:** 1 Pamukkale University Schools of Sport Sciences and Technology, Denizli, Turkey

**Keywords:** Isokinetic strength, anaerobic power, vertical jump, sprinting, agility

## Abstract

The purpose of this study was to investigate the relationship between isokinetic knee strength, anaerobic performance, sprinting ability, agility and vertical jump performance in first division basketball players. Twelve male first division basketball players participated in this study. The mean age was 25.1 ± 1.7 yrs; mean body height 194.8 ± 5.7 cm; mean body mass 92.3± 9.8 kg; mean PBF 10.1± 5.1; and mean VO_2_max 50.55 ± 6.7 ml/kg/min *Quadriceps* and hamstrings were measured at 60° and 180°/s, anaerobic performance was evaluated using the Wingate anaerobic power test, sprint ability was determined by single sprint performance (10–30 m), jump performance was evaluated by countermovement (CMJ) and squat jump (SJ) tests and agility performance was measured using the T drill agility test. Quadriceps strength was significantly correlated with peak power at all contraction velocities. However, for mean power, significant correlation was only found between the 60° left and 180° right knee quadriceps measurements. No measure of strength was significantly related to the measurements from/results of field tests. Moreover, strong relations were found between the performance of athletes in different field tests (p< 0.05). The use of correlation analysis is the limitation of the this study.

## Introduction

Basketball is an aerobic-based anaerobic sport (Delextrat and Cohen, 2009; Meckell et al., 2009; Metaxas et al., 2009) which requires high intensity activities such as jumping (for rebounds, blocks and shots), turns, dribbles, sprints, screens and low intensity activities such as walking, stopping and jogging. Frequent stoppages in games allow players to recover between bouts of activity, thus allowing repeated high-intensity spells of play (Drinkwater, 2008). Aerobic capacity is positively associated with recovery during repeated high-intensity bouts (Castagna et al., 2008; Tomlin and Wenger, 2001). Moreover, the high intensity movements of basketball players are closely related to the development of strength, speed and agility (Castagna et al., 2007; Hedrick, 1993; Meckell et al., 2009). During a basketball game, professional players cover about 3500–5000m (Janeira and Maia, 1998). Each player performs about 1000, mainly short, activities lasting around 2 seconds; time motion analysis has shown that these short activities are performed with a different frequency according to the player’s position (Abdelkrim et al., 2007). Explosive strength, take-off power, speed, and agility are abilities that make an important contribution to efficient movement with and without the ball, thus play an important role in basketball technique and tactics (Erculj et al., 2010). The level of these abilities, that is, the motor potential, is most often measured using various motor tests with and without the ball (Colli et al., 1987). In basketball practice, motor tests are the most suitable and applicable because they are implemented in conditions similar to those of training or competition (Erculj et al., 2010).

Assessment of the physical capacities of athletes is one of the most important issues in modern sports, many test used in order that selection procedures, for screening candidates, or to monitor the efficacy of training regimes (Norkowski, 2002). Despite sports performance professionals and sports scientists focus on performance assessment, there are lack of research examining the relationships between various motor skills (Vescovi and McGuigan, 2008). There are a lot of motor skills in different sports, have kinematic, biomechanical and muscular similarities (Bobbert and van Zandwijk, 1999; Zajac, 2002), but examining correlations between these skills has proved elusive (Vescovi and McGuigan, 2008).

Studies investigate relationships between sprinting and muscle strength performance have had different limitations and reported only weak and no relationships so far (Dowson et al., 1998). The examination of only one joint action or type of muscle contraction, or an incomplete investigation of the relationship between strength and sprint performance measures may be reason of this weak correlations (Dowson et al., 1998).

Sports scientists have examined lower limb strength and power are frequently using the isokinetic knee joint test and the vertical jump test; some studies have focused on the correlation between performance in these two motor skills but have reported scattered findings (Iossifidou, 2005). This scattered findings may be due to a number of differences, such as joint angular velocities and the positioning of the participants, affecting muscle length and velocity of contraction, participant characteristics and methods used for calculation power of joint in isokinetic dynamometry (Iossifidou, 2005).

Some studies have attempted to correlate the results of the WAnT and isokinetic tests. Arslan (2005) found that there were significant correlations between explosive leg strength and anaerobic performance. Kin-İşler et al. (2008) reported that isokinetic concentric knee extension strength was significantly correlated with peak and mean power at all contraction velocities (60°, 150°, 240°). However, there was significant correlation only between 240° knee flexion strength and peak power for isokinetic concentric knee flexion.

While some studies have investigated the relationships between isokinetic knee strength, anaerobic performance, sprinting ability, agility, and vertical jump performance in other athletes, an insufficient number of studies have been conducted on basketball players. Therefore the purpose of this study was to investigate the relationship between isokinetic knee strength, anaerobic performance, sprinting ability, agility and vertical jump performance in first division basketball players.

## Methods

### Subjects and Experimental Approach

Twelve male first division basketball players participated in this study. The mean measurements gathered were as follows: age 25.1 ± 1.7 yrs; body height 194.8 ± 5.7 cm; body mass 92.3± 9.8 kg; PBF 10.1 ± 5.1; and VO_2_max 50.55 ± 6.7 ml/kg/min. The subjects were informed about the possible risks and benefits of the study and gave their informed consent to participate in this study, which was approved by the Clinical Research Ethical Committee of Pamukkale University. The study was conducted over a 1-week period, during which the players did not participate in any other training or matches.

On the first day, anthropometric measurements, vertical jump measurements, sprint tests, agility test and shuttle run test were performed respectively. On the third day, players underwent isokinetic leg strength tests. On the fifth day, players performed the Wingate anaerobic test.

### Anthropometric Measurements

Subjects reported to the laboratory at 8:00 a.m. First, body height (cm), body mass (kg), and percentage of body fat (PBF) measurements were taken for each subject. The body height of the basketball players was measured using a stadiometer accurate to within 1 cm (SECA, Germany), while electronic scales (Tanita BC 418, Japan) accurate to within 0.1 kg were used to measure body mass and percentage of body fat (Lohman et al., 1988).

### Anaerobic performance evaluation

The Wingate Anaerobic Test (WAnT) was conducted using a mechanically braked cycle ergometer (834 E, Monark, Vansbro, Sweden). Subjects were seated on the ergometer and adjustments were made to ensure an optimal cycling position. The WAnT was conducted according to the widely accepted recommendations for standardization (Inbar et al., 1996). The WAnT test was administered for 30 seconds with resistance set at 7.5 % of body mass. The WAnT session started with a standardized warm-up of 5 min of cycling at 50 rpm against no load, after which the subjects rested for 5 min. They were then instructed to pedal as fast as they could. When the pedaling rate reached approximately 160–170 rpm the resistance was applied and subjects continued pedaling as fast as possible for 30s. The subjects were verbally encouraged during the test. Peak power (PP) and mean power (MP) was calculated automatically by the Wingate Anaerobic Test computer program. A fatigue index was calculated by using the following equation (Inbar et al., 1996):

Fatigue Index (FI)=(PP)−(MinP)/(MP)×100

PP is the peak power, and MinP is the minimum power that was determined during the WAnT test. WanT test performed according to Kin-İşler et al.( 2008)

### Isokinetic Leg Strength:

Before the isokinetic test, subjects performed a 5-minute warm-up on a cycle ergometer. Measurements were taken using an Isomed 2000 (Ferstl, Germany) isokinetic dynamometer. The test was performed in a seated position; stabilization straps were secured across the trunk, waist, and distal femur of the tested leg. The leg extensor and leg flexor muscle of each leg were concentrically measured at 60° x s^−1^ (10 repetitions) and 180° x s^−1^ (10 repetitions). Verbal encouragement was given to the subjects during the measurement. Before starting the test, subjects were allowed 5 trials.

### Vertical Jump Measurements

Vertical jump performance was measured using a portable force platform (Newtest, Finland). Players performed countermovement (CMJ) and squat jumps (SJ) according to the protocol described by Bosco et al. (1983). Before testing, the players performed self-administered submaximal CMJs and SJ (2–3 repetitions) as a practice and specific additional warm-up. They were asked to keep their hands on their hips to prevent any influence of arm movements on the vertical jumps and to avoid coordination as a confounding variable in the assessment of the leg extensors (Bosco et al., 1995). Each subject performed 3 maximal CMJs and SJs, with approximately 2 minutes’ recovery in between. Players were asked to jump as high as possible; the best score was recorded in centimeters (Bosco et al., 1995).

### 10–30m Sprint Test

The subjects performed 2 maximal 30 m sprints (with 10 m split times also recorded) on the basketball court. There was a recovery period of 3 minutes between the 30 m sprints. Prior to each sprint test, players performed a thorough warm-up consisting of 10 minutes of jogging at 60–70% of HRmax and then 5 minutes of exercise involving fast leg movements (e.g., skipping, cariocas) over short distances of 5 to 10 m and 3–5 single 15 m shuttle sprints with 2 minutes of passive recovery. Times were measured using an electronic timing system (Prosport TMR ESC 2100, Tumer Engineering, Ankara, Turkey).

### T-Drill Agility Test:

**Figure 1:**
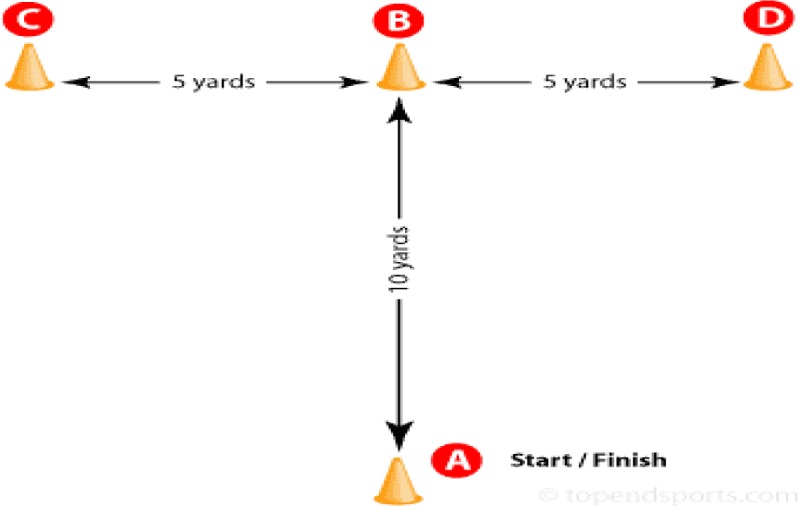
T drill test

Four 22.86 cm collapsible agility cones were arranged as outlined in Semenick (Semenick, 1990) (Figure 1). At the tester’s signal, subjects sprinted forward 9.14 m and touched the tip of the cone (B) with their right hand. Then they performed a lateral shuffle to the left 4.57 m and touched the tip of the cone (C) with the left hand. Subjects then changed direction and shuffled 9.14 m to the right to touch the tip of the cone (D) with their right hand.

They then shuffled 4.57 m to the left to touch point (B) with their left hand. Finally, the subjects back-peddled 9.14 m, passing through the finish at point A (Patterson et al., 2008). Times were measured using an electronic timing system (Prosport TMR ESC 2100, Tumer Engineering, Ankara, Turkey).

### Multi-Stage 20m Shuttle Run Test

Subjects’ maximal oxygen uptake (VO_2_max) was indirectly obtained using a multi-stage 20 m shuttle run test. This consisted of shuttle running between two parallel lines set 20 m apart, running speed cues being indicated by signals emitted from a commercially available pre-recorded audiocassette tape. The audiocassette tape ensured that subjects started running at an initial speed of 8.5 km × h^−1^ and that running speed increased by 0.5 km × s^−1^ each minute. This increase in running speed is described as a change in test level. The speed of the cassette player was checked for accuracy in accordance with the manufacturer’s instructions before each application. All subjects performed a 10-minute warm up that included the prescribed jogging and stretching. Test results for each subject were expressed as a predicted VO_2_max obtained by cross-referencing the final level and (completed) shuttle number at which the subject became exhausted with that of the VO_2_max table provided in the instruction booklet accompanying the multi-stage 20 m shuttle run test. Only fully completed 20 m shuttle runs were considered (Léger and Gadoury, 1989).

### Statistical Analyses

The relationships between isokinetic knee strength, anaerobic performance, sprinting ability, agility and vertical jump performance were evaluated using Pearson Product Moment Correlation analysis. All analysis were executed in SPSS for Windows version 17.0 and the statistical significance was set at p < 0.05.

## Results

The anaerobic performance, isokinetic knee strength, sprint, agility, vertical jump and aerobic power measurements of the basketball players in the study are displayed in Tables 1, 2 and 3, respectively.

Table 4 shows the correlations between anaerobic performance and isokinetic knee strength are presented. isokinetic concentric knee extension strength was significantly correlated with peak power at all contraction velocities. However, for mean power, a significant correlation was only found between the 60° left and 180° right knee extension strength and mean power.

There was no significant correlation between knee strength and field tests.

There was a significant but weak correlation between PP, CMJ, SJ and 10 m sprint performance (Table 5). Also strong correlations were found between the field tests, as it can be seen in Table 6.

## Discussion

One of the important finding of the present study was that there were no relation between any measure of strength and single sprint performance. Similarly, Cronin and Hansen (2005) and Kin-İşler et al. (2008) determined no relation between extension strength and knee flexion and single-sprint performance. No relation were reported between strength measures and 10 or 40 m sprint performance in rugby players by Baker and Nance (1999). On the other hand, Dowson et al. (1998) found a statistically significant relationship between concentric and eccentric knee extensor torques and 0–15 m and 30–35 m sprint times. Similarly, Alexander (1989) found a strong correlation between sprint performance, 100 m personal best sprint time and concentric knee extension torque, at 4.14 rad s^−1^, in elite sprinters. Newman and colleagues (2004) reported a significant correlation between concentric isokinetic knee extension and flexion strength measures and single-sprint performance in football players. One of plausible explanation for the lack of association between isokinetic knee strength and single-sprint performance could be due to the particular characteristics of the subjects (Kin-İşler et al. 2008). Body height is very important for basketball players, particularly for centers and might be one of the reasons for not finding an association between strength and single sprint performance. Another important factor may be the different distances were used in sprint tests in previous studies.

We found a significant relationship between 10 m PP and 10 m sprint ability but no significant relationship between the other Wingate test results and sprint ability. Strong negative correlations (ranges from −0.67 to −0.91) have been demonstrated between performance in the WAnT and sprint speed by previous studies (Kaczkowski et al., 1982; Tharp et al., 1985; Patton and Duggan, 1987). These studies suggested that the WAnT may be used as a predictor of sprinting ability. However, the predictive ability of the WAnT may be related to the distance of the sprint. Sprint times for distances of 37 or 46 m have been reported to be highly correlated with PP, while increased sprint distances appear to be better correlated with MP (Hoffman et al., 2000).

A Further finding of this study is that of a significant relationship between quadriceps strength and PP. This result is similar to findings of previous studies. For instance, Kin-İşler et al. (2008) stated that there was a significant relationship between anaerobic power, capacity and peak isokinetic concentric knee extension strength at all contraction velocities (60°, 150°, 240°). Similarly, Baker and Nance (1999b) investigated the correlation between strength and power. They also reported a strong positive correlation between maximum strength and maximum power in rugby players. In another study, Thorland et al. (1987) found a strong significant correlation between anaerobic power and capacity and isokinetic knee strength in female middle-distance runners and sprinters. Moreover, Mayhew et al. (2001) and Arslan (2005) found that peak and mean power were correlated with explosive leg strength.

No significant relationship was found between strength and field test performances. The weak relationship in rank order of performance could be caused by the differences type of exercise (Hoffman et al, 2000). Hoffman et al, ‘stated that ‘differences in power produced with the legs acting simultaneously or successively, or when upper-body musculature is active or passive, may have a profound influence on power expression (Tharp et al. 1985)’.

The result of this study is that performances in a variety of field tests were correlated with each other in a group of basketball players. It can be said that either the tests assess similar attributes or performance on one test is able to predict performance on another (Vescovi and McGuigan, 2008). Cronin and Hansen (2005) reported weak negative associations between countermovement and squat jump performance and 5, 10, and 30 m sprint times. Hennessy and Kilty (2001) found countermovement jump performance was related to the times for sprint tests and that the bounce drop jump index was related with 30 m and 100 m sprint times in a group of female athletes. The relationship between linear sprinting and agility performance have been examined by few studies (Little and Williams, 2005; Paoule et al., 2000; Vescovi and McGuigan, 2008). Moderate correlation was reported between T-test performance and 37 m sprint times in a group of college-aged women by Paoule et al. (2000). In contrast, Little and Williams (2005) found a weak correlation between acceleration (10 m) and maximum speed in a zigzag agility test in a group of professional male soccer players. The association between agility and speed increases with longer distances and when examining agility with flying sprint times (Vescovi and McGuigan, 2008). The reason of differences between studies could be the use of different agility tests (Vescovi and McGuigan, 2008)..

## Conclusion

One possible reason for the lack of correlation between tests performance may be the different energy systems that each measure needs. In isokinetic knee strength, sprint tests and vertical jump test do not last more than 5 seconds. Therefore the phosphagen system (ATP-PC) contributed to the energy need for these tests. On the other hand the Wingate test glycolytic system is dominant to the energy production. Different energetic pathways used during the tests could be the reason for the lack of association between these measures (Kin-İşler et al; 2008)

## Figures and Tables

**Table 1:** Anaerobic performance values of Basketball players

	Mean	Std
Mean Power (W)	702.81	79.26
Peak Power (W)	955.31	117.86
Fatigue Index (%)	54.67	7.34

**Table 2:** Peak isokinetic concentric knee extension and flexion torques of Basketball players

	Mean	Std
Hamstring Left 180°/s (Nm)	130.36	20.94
Hamstring Right 180°/s (Nm)	137.36	25.10
Quadriceps Left 180°/s (Nm)	177.45	30.92
Quadriceps Right 180°/s (Nm)	173.27	25.34
Hamstring Left 60°/s (Nm)	159.18	36.42
Hamstring Right 60°/s (Nm)	163.81	30.05
Quadriceps Left 60°/s (Nm)	207.72	47.75
Quadriceps Right 60°/s (Nm)	203.27	37.03

**Table 3:** Field Performance Test Results of Basketball players

	Mean	Std
VO_2max_ (ml/kg/min)	50.55	6..72
CMJ (cm)	34.91	3.83
SJ (cm)	32.91	3.82
10m (s)	1.86	.30
30m (s)	4.34	.15
T Drill test (s)	9.25	.46
PBF (%)	10.01	5.10

VO_2max_ = Maximal oxygen uptake, CMJ = Counter movement jump, SJ= Squat jump, PBF= Percantage of body fat

**Table 4:** Correlations between anaerobic performance and isokinetic knee Strength

	Peak Power	Mean Power	Fatigue İndex
Hamstring Left 180°/s (Nm)	NS	NS	NS
Hamstring Right 180°/s (Nm)	NS	NS	NS
Quadriceps Left 180°/s (Nm)	.46^*^	NS	NS
Quadriceps Right 180°/s (Nm)	.55^**^	.40^*^	NS
Hamstring Left 60°/s (Nm)	NS	NS	NS
Hamstring Right 60°/s (Nm)	NS	NS	NS
Quadriceps Left 60°/s (Nm)	.57^**^	.51^*^	NS
Quadriceps Right 60°/s (Nm)	.49^*^	NS	NS

* p < 0.05.

** p < 0.01.

**Table 5:** Correlations between anaerobic performance and field test

	Peak Power	Mean Power	Fatigue İndex
VO_2_max (ml/kg/min)	NS	NS	NS
CMJ (cm)	.49^*^	NS	NS
SJ (cm)	.55^**^	.43^*^	NS
10m (s)	.−52^**^	NS	NS
30m (s)	NS	NS	NS
T Drill test (s)	NS	NS	NS
PBF (%)	NS	NS	NS

* p < 0.05.

** p < 0.01.

VO_2max_ = Maximal oxygen uptake, CMJ = Counter movement jump, SJ= Squat jump, PBF= Percantage of body fat

**Table 6:** Correlations between field tests

	CMJ	SJ	10m	30m	T Dtest	BPF (%)
CMJ (cm)		.807^**^	NS	−.619^*^	−.594^*^	−.487^*^
SJ (cm)	807^**^		NS	−.760^**^	−.473^*^	−.494^*^
10m (s)	NS	NS		NS	NS	NS
30m (s)	−.619^*^	−.760^**^	NS		505^*^	NS
T Drill test (s)	−.594^*^	−.473^*^	NS	.505^*^		NS
PBF (%)	−.487^*^	−.494^*^	NS	NS	NS	

* p < 0.05.

** p < 0.01.

VO_2max_ = Maximal oxygen uptake, CMJ = Counter movement jump, SJ= Squat jump, PBF= Percantage of body fat
